# Lesion mimic mutant 8 balances disease resistance and growth in rice

**DOI:** 10.3389/fpls.2023.1189926

**Published:** 2023-06-05

**Authors:** Mengcan Zhao, Yunxia Guo, Hang Sun, Jichao Dai, Ximan Peng, Xuedong Wu, Han Yun, Lisha Zhang, Yiming Qian, Xinying Li, Guanghua He, Changwei Zhang

**Affiliations:** Key Laboratory of Application and Safety Control of Genetically Modified Crops, Rice Research Institute, Southwest University, Chongqing, China

**Keywords:** rice, lesion mimic, protoporphyrinogen IX oxidase, resistance, growth

## Abstract

Lesion-mimic mutants (LMM) spontaneously produce necrotic spots, a process not affected by environmental stress or pathogen infection. In this study, we identified a LMM, *lesion mimic mutant 8* (*lmm8*) in rice (*Oryza sativa*). The *lmm8* mutant produces brown and off-white lesions on its leaves during the second- and third-leaf stages. The lesion mimic phenotype of the *lmm8* mutant was enhanced by light. At the mature stage, *lmm8* mutant are shorter and exhibit inferior agronomic traits than the wild type. Contents of photosynthetic pigments and chloroplast fluorescence were significantly reduced in *lmm8* leaves, along with increased production of reactive oxygen species and programmed cell death compared to the wild type. The mutated gene was identified as *LMM8* (*LOC_Os01g18320*) by map-based cloning. A point mutation occurred in *LMM8*, causing a Leu to Arg mutation of the 146th amino acid of LMM8. It is an allele of *SPRL1*, encoding a protoporphyrinogen IX oxidase (PPOX) located in chloroplasts and involved in the biosynthesis of tetrapyrrole in chloroplasts. The *lmm8* mutant showed enhanced resistance and broad-spectrum resistance. Together, our results demonstrate the importance of rice LMM8 protein in defense responses and plant growth in rice, and provides theoretical support for resistance breeding to improve rice yield.

## 1 Introduction

Tetrapyrrole biosynthesis in plants involves many genes. For instance, twenty-seven genes encoding tetrapyrrole synthetases have been identified in *Arabidopsis thaliana* (Beale, 2005; Li et al., 2019). Rice (*Oryza sativa*) contains 21 genes that are homologous to these Arabidopsis genes and thus are candidates to encode tetrapyrrole synthetases (Ge et al., 2014). Among these candidates, 14 genes have been identified and cloned. Like heme, siroheme, and phytochromobilin, chlorophyll is a tetrapyrrole. Chlorophyll, an important photosynthetic pigment, is vital for plant growth and development (Tanaka and Tanaka, 2007; Jiang et al., 2022). So, the proteins encoded by these genes are important for the synthesis of the tetrapyrrole. During plant development, if they mutated, the synthesis of tetrapyrrole in plants will be defective, which will lead to a variety of different color mutations in plant leaves, and eventually lead to slow growth and decreased yield (Bruggeman et al., 2015).

Intriguingly, mutants for several of these genes show lesion-mimic phenotypes. For example, *Rice Lesion Initiation 1* (*RLIN1*) encodes a putative coproporphyrinogen III oxidase in the tetrapyrrole biosynthesis pathway that catalyzes the conversion of coproporphyrinogen III to protoporphyrinogen IX, the *rlin1* mutant exhibits a lesion-mimic phenotype (Sun et al., 2011). *Oryza sativa Protochlorophyllide Oxidoreductase A* (*OsPORA*) and *OsPORB*, encoding protochlorophyllide oxidoreductase A and B, respectively, catalyze the photoreduction of protochlorophyllide (Pchlide) to chlorophyllide during chlorophyll biosynthesis. The *ospora* mutant shows necrotic spots, with reduced growth and chlorophyll content (Li et al., 2019), and the *osporb* mutant exhibits yellow, necrotic spots during leaf elongation (Sakuraba et al., 2013). *Spotted and Rolled Leaf* (*SPRL1*) encodes a protoporphyrinogen IX oxidase that can catalyze the synthesis of protoporphyrin IX from protoporphyrinogen IX, the *sprl1* mutant exhibits spotted and rolled leaf (Liu et al., 2022).

Lesion-mimic phenotypes can be caused by environmental factors, accumulation of reactive oxygen species (ROS), disordered metabolic pathways, programmed cell death (PCD), or phytohormone imbalance. Notably, lesion-mimic mutants often show enhanced resistance to pathogenic bacteria. For example, *cell death and resistance 1* (*cdr1*) (Takahashi et al., 1999), *Oryza sativa lesion mimic and senescence* (*oslms*) (Undan et al., 2012), and *tos17 triggered mutation1* (*ttm1*) (Takahashi et al., 2007) are resistant to rice blast, and *spotted leaf 19* (*spl19*) (Wu et al., 2008), *Oryza sativa non-expressor of pr1* (*osnpr1)* (Chern et al., 2005), and *spotted leaf 30* (*spl30*) (Xu et al., 2014) are resistant to bacterial blight. Some lesion-mimic mutants, such as *spotted leaf 11* (*spl11*) (Sanchez and Khush, 2000), *chloroplastic-H_2_O_2_-induced lesion* (*chl1*) (Han et al., 2014) and *spotted leaf 28* (*spl28*) (Qiao et al., 2010), show resistance to both rice blast and bacterial blight. *Oryza sativa lsd1* (*oslsd1*) is resistant to blast and the fungal toxin fumonisin B1 (Wang et al., 2005), and *lesion mimic mutant 1* (*lmm1*) is resistant to rice blast and banded sclerotial blight (Wang et al., 2007). Notably, *leaf lesion mimic mutant 1* (*llm1*) (Wang et al., 2015), which is allelic to *rlin1*, exhibits enhanced resistance to bacterial blight due to defective coproporphyrinogen III oxidase, an enzyme that is indispensable for tetrapyrrole biosynthesis.

Chlorophyll, heme and phytochromobilin are tetrapyrrole derivatives that are produced from protoporphyrin IX, which is generated from glutamate. Protoporphyrin IX binds with different ions to form different pyrrole compounds, including Mg^2+^ to form chlorophyll and Fe^3+^ to form heme and phytochromobilin (Tanaka and Tanaka, 2007). Protoporphyrinogen IX oxidase (PPOX), which oxidizes the conversion of protoporphyrinogen IX to protoporphyrin IX, is an important enzyme in tetrapyrrole biosynthesis. Defects in PPOX can damage plants. PPOX has been studied in many species, such as *Arabidopsis* (Zhang F. et al., 2014), humans (Wang et al., 2021), tobacco (*Nicotiana tabacum*) (Lermontova et al., 1997), yeast (*Saccharomyces cerevisiae*) (Nishimura et al., 1995), and *Escherichia coli* (Sasarman et al., 1993). PPOX in rice has only been studied in terms of its role in herbicide resistance (Liu et al., 2022), little is known about the role of this enzyme in the defense response.

To further elucidate mechanisms of lesion mimic or spontaneous programmed cell death in rice, we isolated a LMM named *lmm8* (*lesion mimic mutant 8*) in rice. Brown and off-white lesions were always present in *lmm8* beginning at the second-leaf stage, and the lesion mimic phenotypes could be induced by light. Compared to the wild type, the plant type of *lmm8* was negatively affected. The *lmm8* mutant showed excessive reactive oxygen species (ROS) accumulation and increased programmed cell death (PCD) in its leaves, perhaps leading to the lesion-mimic phenotype. Gene mapping and complementation experiments showed that *LMM8* is the *LOC_Os01g18320* gene, which encodes a PPOX. LMM8 localizes to the chloroplast, and *LMM8* is mainly expressed in leaf mesophyll cells. Compared to the wild type, the *lmm8* mutant showed enhanced, broad-spectrum resistance to rice blast strains and bacterial blight, indicating that *LMM8* induces defense responses in rice. Therefore, our results suggest that *LMM8* functions in plant resistance and plant growth in rice. It provides a new clue for the further study of the function of LMM8.

## 2 Materials and methods

### 2.1 Materials

The mutant, designated *lmm8* (it is the 8th lmm identified by this group), was screened from an EMS mutagenized population of the indica rice maintainer line ‘Xinong 1B’ (wild type; WT). After repeated multi-generation selfing, the mutant phenotype was inherited stably. The F_1_ generation plants were obtained by crossing indica rice restorer line Jinhui 10 (J10) with *lmm8*, and the F_2_ generation plants obtained by F_1_ selfing were used for gene mapping analysis. There are differences in the genome between J10 and Xinong 1B, which is convenient to design polymorphic molecular markers in gene mapping experiment. All materials were planted in the experimental field of Southwest University, Chongqing, China.

### 2.2 Analysis of main agronomic characters

In mid-March, mutant and wild type seeds were germinated in the incubator to the second leaf stage and subsequently transplanted to the field. Pictures of the leaf phenotype were taken for each period. At maturity, ten wild type and *lmm8* mutant plants were randomly selected to investigate plant height, effective panicle number, panicle length, grain number per panicle, seed setting rate, 1000-grain weight and other agronomic traits.

### 2.3 The determination of photosynthetic characteristics

At the heading stage, the same parts of flag, second and third leaves of wild type and *lmm8* mutant were selected to analyze the photosynthetic characteristics. At 10:00 AM on sunny days, the net photosynthetic rate (Pn), stomatal conductivity (Gs), intercellular CO_2_ concentration (Ci), and transpiration rate (Tr) were measured by Li-6800 convenient photosynthetic tester (LICOR, USA) with three repetitions. The photosynthetic pigment contents in the flag, second and third leaves of wild type and *lmm8* mutant at heading stage were determined by previous method (Lichtenthaler and Babani, 2022). Briefly, from 8:30 to 9:00 AM, 0.05g leaves were cut into pieces and soaked in 25 mL ethanol and acetone solution (V/V=1:1). Then, the samples were treated in the dark for 24 h with several oscillations. The absorption at 663 nm, 645 nm and 470 nm were measured by a spectrophotometer and the photosynthetic pigment content was calculated. All samples were analyzed with three repetitions.

### 2.4 Frozen section and scanning electron microscopy

The middle portion of the second leaf of wild type and *lmm8* mutant plants sampled at tillering stage was embedded with embedding agent and frozen in liquid nitrogen. The sample was cut into 8 μm-thick sections with a freezing microtome (Thermo Scientific, USA), and rinsed with sterilized water and photographed under the fluorescence microscope(OLYMPUS,18A16537). For scanning electron microscopy (SEM) observation, the intrathecal epidermis of the wild type and the *lmm8* mutant the second leaf was fixed on the conductive adhesive and observed under 250 SE (Secondary Electronic) scanning electron microscopy. At the spike stage, obvious diseased spots in wild type and mutant flag leaves were taken, fixed on the sample table with conductive adhesive, and observed by SEM at 250SE and 700SE.

### 2.5 Histochemical observations and physiological indices

According to previous method, the leaves of wild type and *lmm8* mutant plants at heading stage were stained with trypan blue for cell death detection (Koch and Slusarenko, 1990), with 3,3-diaminobenzidine (DAB) for hydrogen peroxide (H_2_O_2_) detection (Takahashi et al., 1999). For physiological indices, wild type and *lmm8* mutant plant were selected to determine the content of hydrogen peroxide (H_2_O_2_) and the activities of superoxide dismutase (SOD), peroxidase (POD) and catalase (CAT) with commercial kits (Nanjing Jiancheng Technology Co., Ltd, Nanjing, China).

### 2.6 TUNEL analysis

Fresh wild type and *lmm8* mutant leaves were fixed with FAA solution (45% ethanol, 45% ddH_2_O, 5% glacial acetic acid, 5% formaldehyde) for 48 h, dehydrated with different concentrations of ethanol (50%, 70%, 85%, 95%, 100%, 100%, 100%), transparent with different proportions xylene and ethanol mixed solution (V/V=1:3, 1:1,3:1). Then all samples were embedded in paraffin. 8 μm section was cut and performed with TUNEL analysis. The method was referred to the DeadEnd™ Fluorometric TUNEL System kit (Promega, USA).

### 2.7 Cloning of *LMM8*

For the cloning of *LMM8*, the *lmm8* mutant was crossed with Jinhui 10, and mutant plants in the F_2_ population were selected for gene mapping. Simple sequence repeat (SSR) markers from a public rice database (http://www.gramene.org) were used. Furthermore, the insertion/deletion markers were developed by comparing the genomic sequences of Xinong 1B with those of Jinhui 10 in our laboratory. In order to obtain the complementary transgenic plants, a 6342 bp (2256 bp upstream, 3241 bp gene part, 845 bp downstream) sequence of *LMM8* was amplified from the wild type gDNA (primer:F1, R1) and ligated into pCAMBIA1301 vector, then this complementary vector was transformed into *lmm8* mutant using an *Agrobacterium*-mediated approach (You et al., 2022). The primer sequences used for gene cloning and complementary vector are shown in **Supplementary Data 1**.

### 2.8 Protoporphyrin IX content determination

The flag leaves of wild type and mutant plants at heading stage (0.3 g) with liquid nitrogen were ground into homogenate, diluted to 10 ml with 80% acetone, and centrifuged at 13000 g for 10 min. Using 80% acetone as control, the absorbance values at 575 nm, 590 nm and 628 nm were measured. The calculation formula is as follows:


Proto IX=562[0:18016(A575)−0:04036(A628)−0:04515(A590)]×V/(1000×W)

### 2.9 Multiple sequence alignment and evolutionary analysis

A multiple sequence alignment of LMM8 was obtained in NCBI (https://www.ncbi.nlm.nih.gov/) website and ClustalX software. The phylogenetic tree of LMM8 homologous protein was constructed with MEGA software (Species and number were found in the **Supplementary Data 2**).

### 2.10 Subcellular localization

The full-length coding sequence of *LMM8* without stop codon was amplified and inserted into pAN580 vector to obtain the LMM8-GFP fusion protein. Then the vector was transfected into rice protoplasts. After overnight incubation at 28°C, the fluorescent signal of GFP was observed by confocal laser microscopy (Han et al., 2009).

### 2.11 *In situ* hybridization

A 374bp-specific sequence of *LMM8* was amplified from the wild type cDNA and was used for probe preparation with DIG. The sections were then pretreated, hybridized and immunologically tested with previous method (Chen et al., 2018).

### 2.12 Shading and dark germination treatments

In shading treatment, wild type and *lmm8* mutant seed were grown in an incubator (28°C, 70% relative humidity, 20000 Lux, lighting to the second leaf stage), and then half of them were treated in shading for 5 days, and the other half of them served as the control. In dark germination, wild type and *lmm8* mutant seed were germinated and grown in an incubator (28°C, 70% relative humidity) under light (20000 Lux) intensity and dark conditions, respectively.

### 2.13 Inoculation with bacterial leaf blight and rice blast

Leaf blight strain xoo-p6 was donated by Sichuan Agricultural University and incubated in a potato solid medium (potato 300 g/L, tryptone 5 g/L, sucrose 15 g/L, Ca(NO_3_)_2_·4H_2_0 0.5 g/L, Na_2_HPO_4_·12H_2_O 2.0 g/L, agar 20 g) for two days in 28°C under dark conditions. A single pale yellow spot was selected and grown in potato liquid medium (potato 300 g/L, tryptone 5 g/L, sucrose 15 g/L, Ca(NO_3_)_2_·4H_2_0 0.5 g/L, Na_2_HPO_4_·12H_2_O 2.0 g/L) for 14 hours at 28°C and 150 rpm. The bacterial cells were collected with centrifuging and resuspended with sterile water to OD_600_ = 0.5. At the initial tillering stage, the tip of flag leaf was cut about 1cm and dipped with bacterial solution. The lesion length was measured after inoculation 5, 10 and 15 days. The photograph was taken on the 15^th^ day.

The resistance of rice to seedling blast and spike blast was identified in greenhouse and field. All methods followed a previous report (Huang et al., 2000). Briefly, 108 single-spore strains were isolated by single-spore, conidial culture and conidial suspension preparation. The resistance spectrum of rice blast and the resistance identification of seedling blast and spike blast were carried out following (Zhang et al., 2011). The classification and naming of rice blast bacteria were conducted according to the national joint test group of rice blast physiological bacteria (China National Coorporation of Research on Physiological Races of *Pyricularia oryzae*, 1980).

### 2.14 Gene expression analysis

RNA of wild type and *lmm8* mutant was extracted with KKFast Plant RNAPURE Kit (Zhuangmeng biology, China) and reverse transcribed with RT MIX with DNase Kit (Yuyi Landi biology, China). After diluting 20-fold, RT-qPCR(reverse transcription quantitative PCR) analysis was performed using the CFX Connect system (Bio-Rad, USA) and SYBR Premix Ex Taq II kit (TaKaRa, Japan) with 3 three biological repetitions. All primers for relevant gene expression were designed according to the gene sequences in the Gramene database (http://www.Gramene.org/) and *OsActin1* as internal reference gene.

### 2.15 Statistical analysis

GraphPad Prism 8.0.2 (http://www.graphpad.com/) was used for the statistical analysis. Student’s *t*-test was used to examine the experimental data.

## 3 Results

### 3.1 Phenotypic analysis of *lmm8* mutant

**Figure 1:**
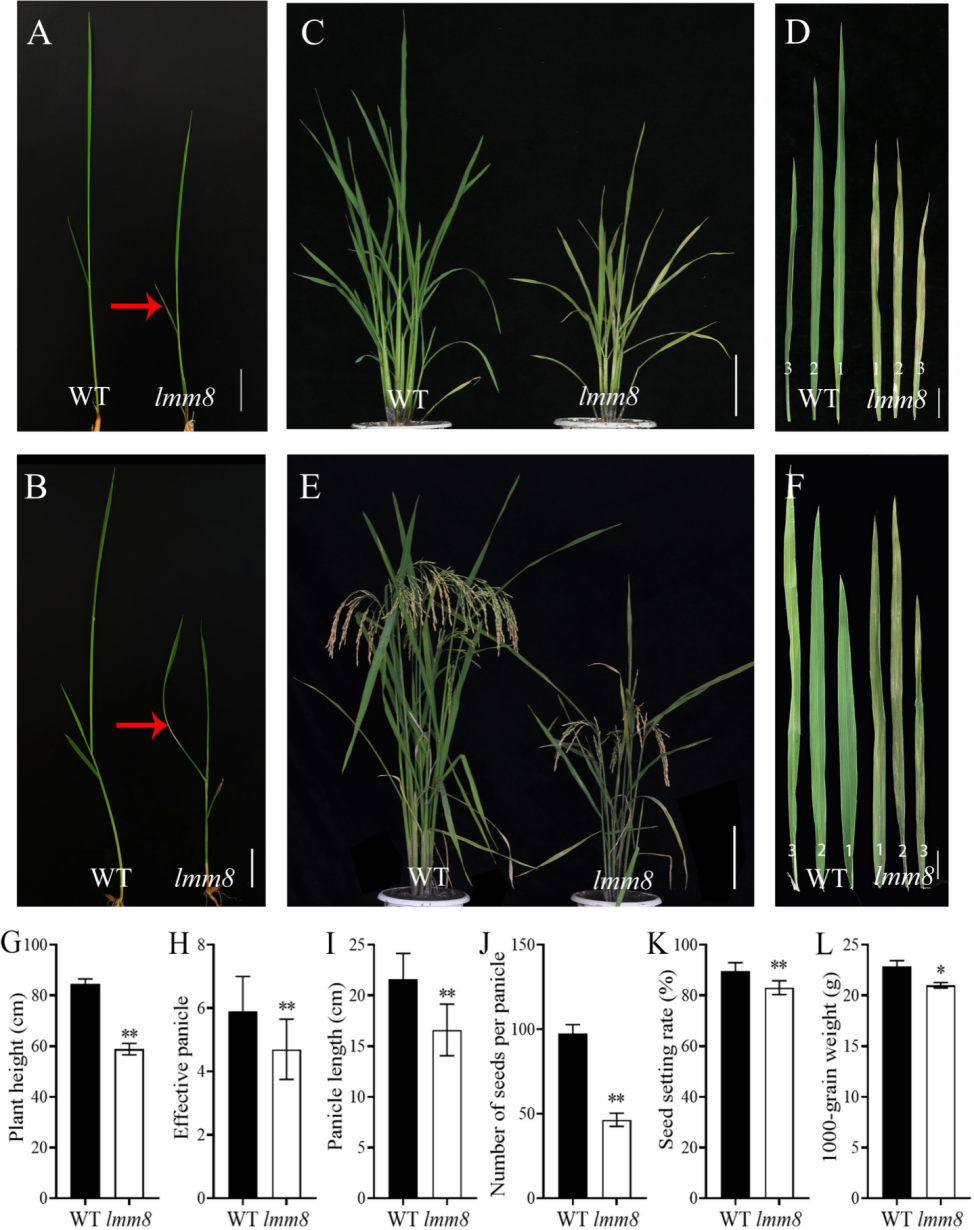
Comparison of phenotypes between wild type and *Imm8* mutant. **(A)** Wild type and *Imm8* mutant plants at two-leaf stage; **(B)** Wild type and *Imm8* mutant plants at three-leaf stage; (red arrows indicate the location of the diseased spots) **(C)** Wild type and *Imm8* mutant plants at tillering stage: **(D)** The first, second and third leaves of wild type and *Imm8* mutant at tillering stage: **(E)** Wild type and *Imm8* mutant plants at mature stage; **(F)** The first, second and third inverted leaves of wild type and *Imm8* mutant pants at mature stage; **(A, B)** scale bars = 2 cm; **(C, E)** scale bars = 10 cm; **(D, F)** scale bars = 4 cm, **(G–L)** Main agronomic traits of the wild type and *Imm8* mutants; values represent means ± SD (n = 10); (*: *P* < 0.05, ** : *P* < 0.01).

Under the same growth conditions, the oldest leaves of the *lmm8* mutant showed clear brown streaks beginning at the second-leaf stage, while wild type leaves appeared normal (**Figure 1A**). At the third-leaf stage, grayish-white leaves were observed in *lmm8* (**Figure 1B**). Moreover, *lmm8* plants were shorter than the wild type. At the late tillering stage, some small brown patches appeared on the grayish-white leaves of the mutant (**Figures 1C, D**). Based on this phenotype, we named this mutant *lesion mimic mutant 8* (*lmm8*).

**Figure 2:**
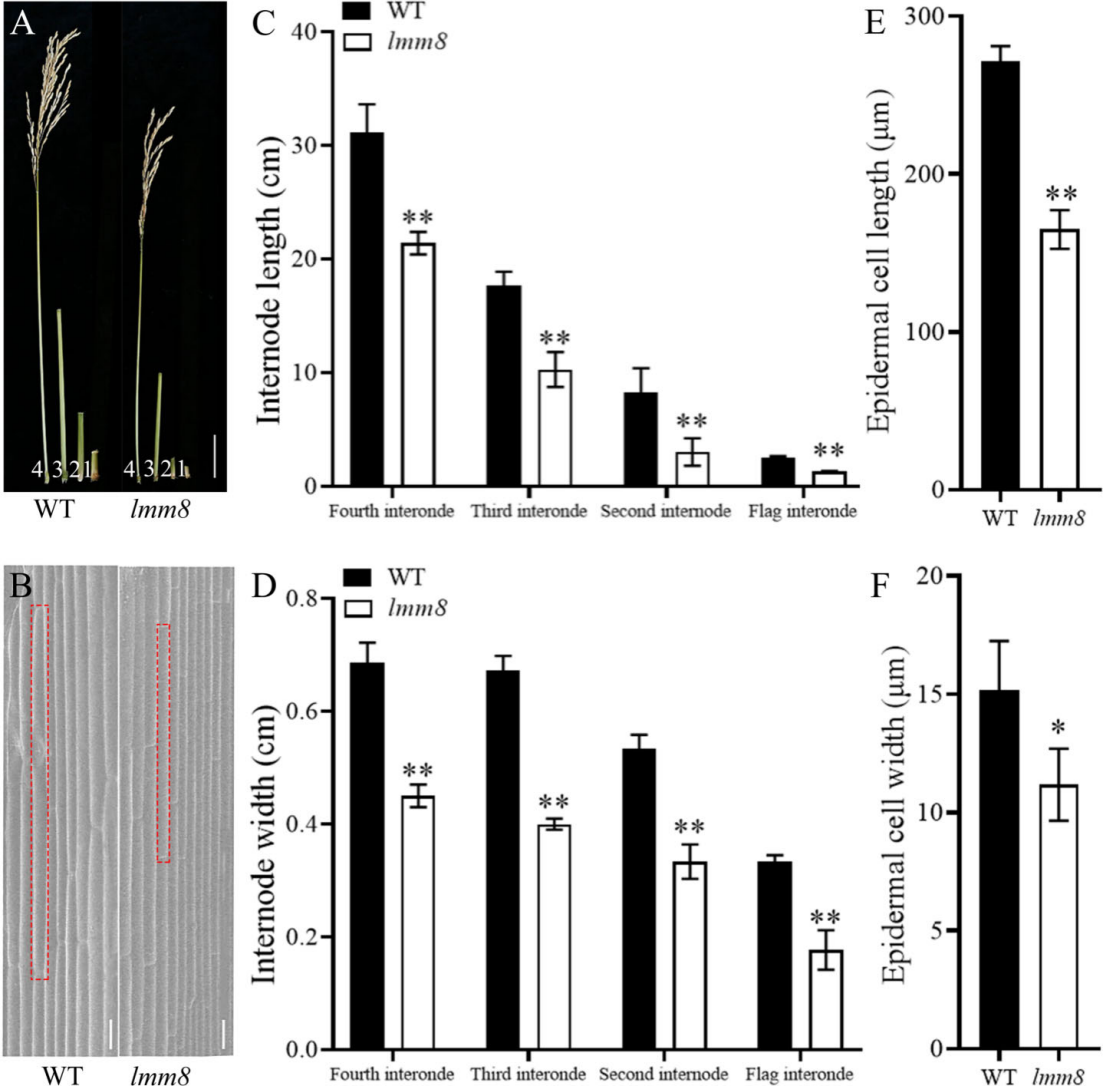
Analysis of dwarfing phenotype in *Imm8* mutant. **(A)** Flag internodes, second internodes and third internodes at mature stage (labeled 1, 2,3 and 4 in turn), scale bar=5cm; **(B)** Scanning electron microscopy of leaf sheath epidermal cells of wild type and *Imm8* mutant, scale bar = 10μm: **(C)** The internode length of wild type and *Imm8* mutant: **(D)** The internode width of wild type and *Imm8* mutant; values represent means ± SD (n=10); **(E)** Cell length of wild type and *Imm8* mutant leaf sheath epidermal; **(F)** Cell width of wild type and *Imm8* mutant leaf sheath epidermal (n=3); (*: *P* < 0.05, **: *P* < 0.01).

As the temperature increased during the mature stage, the lesion-mimic area of the same leaf was smaller than that at the seedling or tillering stage, and *lmm8* plants showed dwarfing, shrinkage, and shortening compared to wild type plants (**Figures 1E, F**). The plant height, panicle length, grain number per panicle, seed setting rate, effective panicle number, and 1000-grain weight of the *lmm8* mutant were much lower than those of the wild type (**Figures 1G–L**). We next measured the seed length and width and found the seed width with little difference in grain width, butthe *lmm8* mutant grain length was significantly reduced relative to that of the wild type (**Figure S1**). In addition, internodes length and width of the *lmm8* mutant were significantly shorter than wild type internodes (**Figures 2A, C, D**). Because the *lmm8* mutant was obviously shorter than the wild type beginning at the seedling stage, we examined the leaf sheaths of wild type and *lmm8* plants at the seedling stage. The length and width of the inner epidermal cells were strongly reduced in the *lmm8* mutant compared to the wild type (**Figures 2B, E, F**), suggesting that *lmm8* cells are smaller, leading to plant dwarfing. Therefore, the mutation of *LMM8* affected many aspects of rice, such as leaf size, panicle type, plant height, and yield, indicating that LMM8 plays an important role in rice development.

### 3.2 *lmm8* leaves show reduced photosynthetic capacity

**Figure 3:**
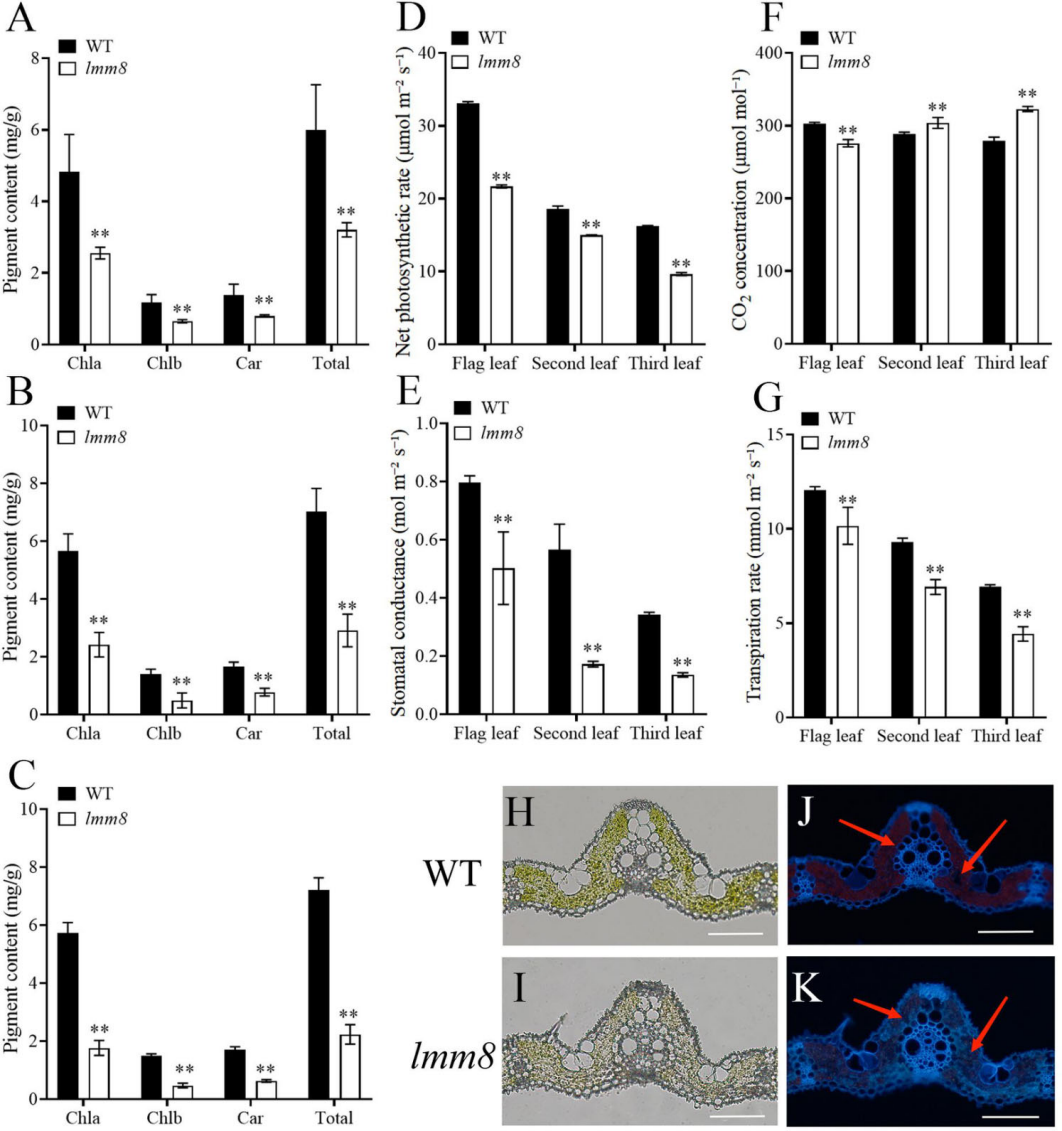
The chlorophyll autofluorescence, photosynthetic pigment and characteristics analysis between the wild type and *Imm8* mutant. **(A–C)** The content of photosynthetic pigment (chlorophyll a, chlorophyll b. Carotenoid and total chlorophyll) in the flag **(A)**, second **(B)** and third **(C)** leaves of wild type and *Imm8* mutant: **(D–G)** The net photosynthetic rate **(D)**, stomatal conductance **(E)**, intercellular CO_2_ concentration **(F)** and transpiration rate **(G)** of the flag, second and third inverted leaves of wild type and *Imm8* mutant: values represent means ± SD (n = 3); (*: *P* < 0.05. **: *P* < 0.01). **(H, I)** The cross-section of wild type and *imm8* mutant leaf under microscope bright field: **(J, K)** The cross-section of wild type and *Imm8* mutant leaf under fluorescence (The red arrows indicate the chloroplast autofluorescence), scale bars = 100μm.

The defective leaves of the *lmm8* mutant suggested that biosynthesis of photosynthetic pigments might be impaired in this mutant. We therefore extracted photosynthetic pigments from *lmm8* and wild type leaves and measured their levels. Compared to the wild type, the contents of chlorophyll a, chlorophyll b, carotenoid, and total chlorophyll in the flag, second and third leaves at the heading stage were significantly lower in the *lmm8* mutant than the wild type (**Figures 3A–C**). In addition, the net photosynthetic rate and stomatal conductance were significantly reduced in *lmm8* leaves vs. the wild type (**Figures 3D, E**). The intercellular CO_2_ concentration of the flag leaf was significantly lower in *lmm8* than the wild type, while the intercellular CO_2_ concentrations of the second and third leaves were significantly higher in *lmm8* than the wild type (**Figure 3F**). Finally, the transpiration rate was significantly lower in *lmm8* leaves than the wild type (**Figure 3G**). These results suggest that *lmm8* leaves have reduced photosynthetic capacity.

To test this notion, we examined frozen sections of leaf tissue under a microscope. Although there was no significant difference in leaf structure between the *lmm8* mutant and wild type (**Figures 3H, I**), the red fluorescence intensity generated by the autofluorescence of chloroplasts was significantly weaker in the *lmm8* mutant than the wild type (**Figures 3J, K**), suggesting the *lmm8* chloroplasts were seriously damaged.

### 3.3 Analysis of ROS accumulation and PCD in *lmm8* leaves

**Figure 4:**
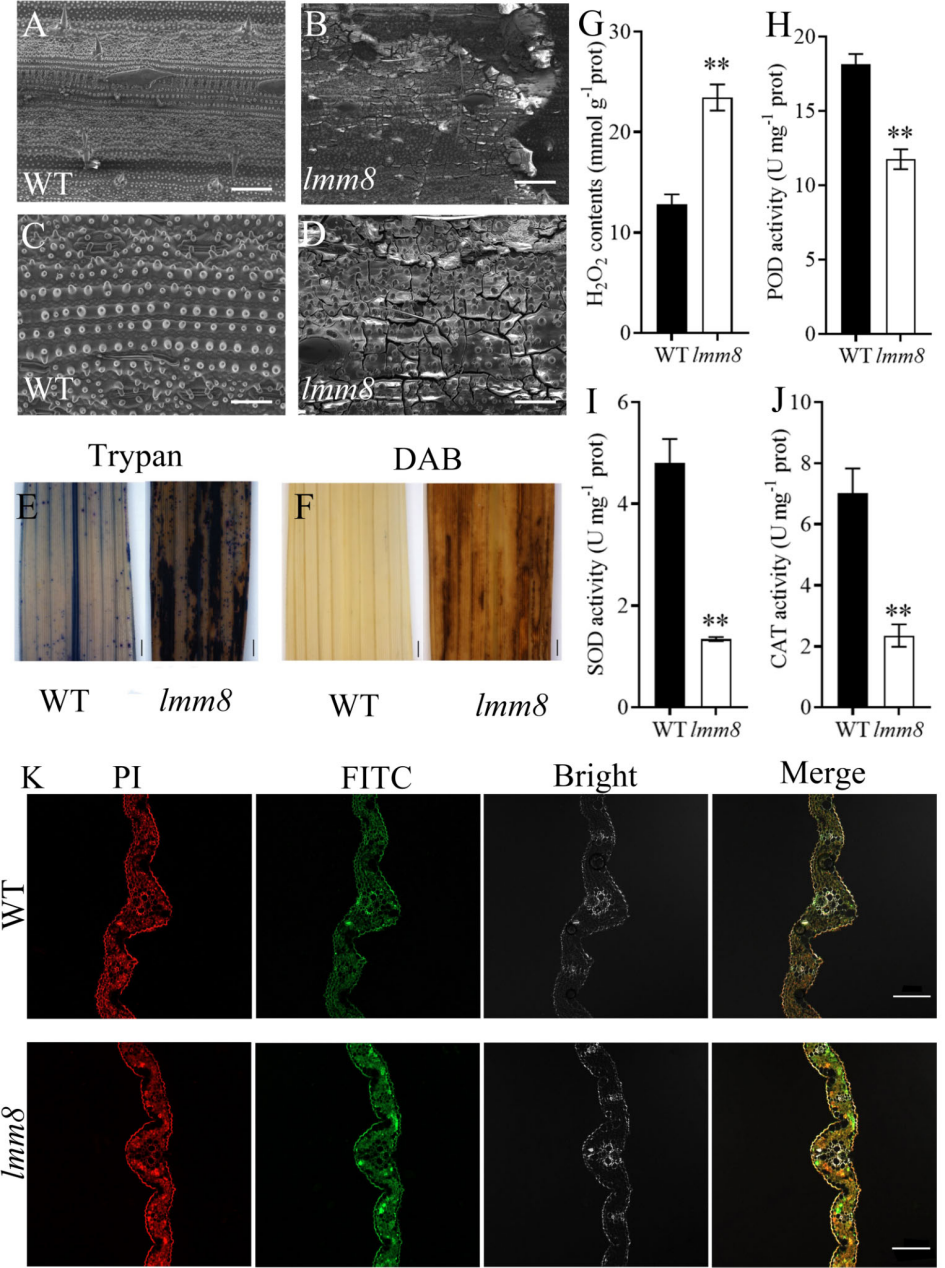
Analysis of ROS accumulation in wild type and *Imm8* mutant leaves and the PCD analysis of leaves **(A, B)** 250 × SEM of tillering stage leaves of wild type and *Imm8* mutants; scale bars= 200μm. **(C, D)** 700 × SEM of tillering stage leaves of wild type and *Imm8* mutants; scale bars = 50μm. **(E)** Trypan blue staining of wild type and *Imm8* mutant leaves; **(F)** DAB staining of wild type and *Imm8* mutant leaves: **(E, F)** scale bars= 1 mm; **(G–J)** The content of hydrogen peroxide (H_2_O_2_) activity of Peroxidase (POD). Superoxide dismutase (SOD) and Catalase (CAT) wild type and *Imm8* mutant leaves; values represent means ± SD (n = 3) (*: *P* < 0.05, **: *P* < 0.01); **(K)** TUNEL staining of wild type and *Imm8* mutant leaves (Propidium iodide staining is indicated by red fluorescence and TUNEL positive staining is indicated by yellow to green fluorescence), scale bars = 100μm.

Both the inner and outer epidermal structures of wild type leaves were clearly visible, but at the lesion-like sites of *lmm8* leaves, many cells were broken, as revealed by scanning electron microscopy (**Figures 4A–D**). Since cell damage in plants is closely associated with ROS accumulation (Zhang M. et al., 2014), we performed trypan blue and DAB (diaminobezidin) staining of wild type and *lmm8* leaves to measure ROS accumulation. Compared to the wild type, a large amount of trypan blue staining and reddish-brown deposits (DAB staining) were observed in the lesion-like spots of *lmm8* leaves (**Figures 4E, F**).

We then measured H_2_O_2_ contents and the activities of the H_2_O_2_-scavenging enzymes CAT, peroxidase (POD), and superoxide dismutase (SOD) in wild type and *lmm8* leaves of plants at the heading stage. The *lmm8* mutant had significantly higher H_2_O_2_ contents than the wild type, indicating that more H_2_O_2_ accumulated in *lmm8* (**Figure 4G**), which is consistent with the results of DAB staining. In addition, CAT, POD, and SOD activities were significantly lower in the *lmm8* mutant than the wild type (**Figures 4H–J**), indicating that the active oxygen scavenging ability was reduced in the *lmm8* mutant. These results indicate that more ROS accumulated in *lmm8* leaves than the wild type. Excessive ROS induces PCD (programmed cell death) in plant cells. To further verify PCD in the lesion-like structures of *lmm8*, we performed a TUNEL analysis. Wild type leaves showed a faint green fluorescence signal, whereas a strong green fluorescence signal was detected in *lmm8* leaves (**Figure 4K**), suggesting that PCD occurred in the mutant leaves.

### 3.4 Localization and cloning of the candidate gene

**Figure 5:**
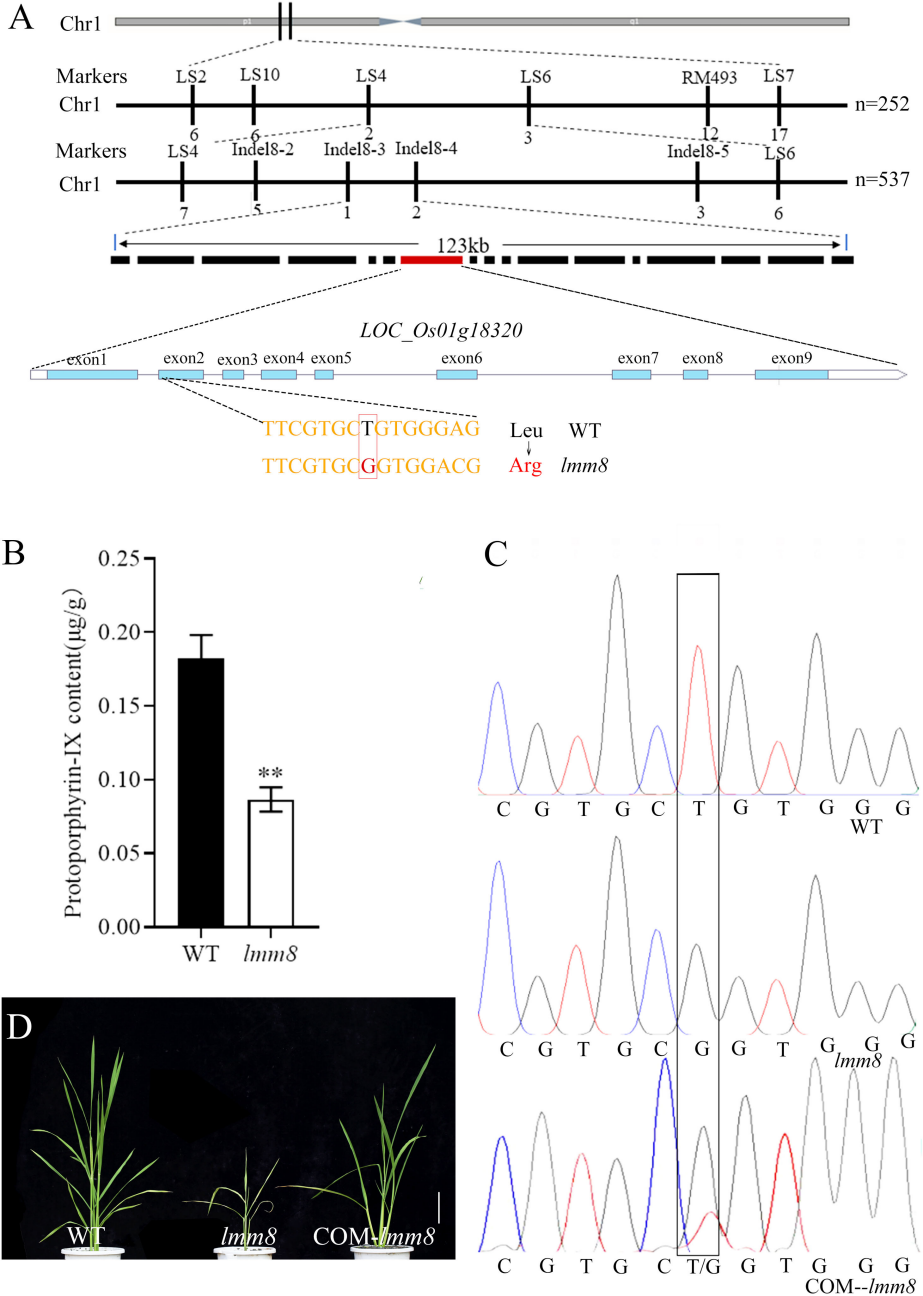
Cloning of *LMM8* and analysis of the PPOX enzyme activity. **(A)** Gene mapping of *LMM8*; **(B)** Protoporphyrin-IX content of wild type and *Imm8* mutant; values represent means ± SD (n = 3) (*: *P* < 0.05, **: *P* < 0.01) **(C)** Sequenceding the mutated site in the wild type, *Imm8* mutant and T_0_ transgenic plant; **(D)** Functional complementation of mutant *Imm8:* scale bar = 10 cm.

We crossed the *lmm8* mutant with the *indica* restorer line ‘Jinhui 10’ and examined the progeny. Plants of the F_1_ generation showed no lesion-like spots, whereas various plants in the F_2_ generation derived from self-crossing of F_1_ plants exhibited this trait. We counted plants with mutant and normal phenotypes. Among the 2320 plants examined, 1762 plants appeared normal and 558 showed lesion-like spots. Chi-square test results (χ^2^ = 1.11 < χ^2^_0.05_ = 3.84) matched a Mendelian segregation ratio of 3:1, indicating that the mutant phenotype is genetically stable and controlled by a single recessive nuclear gene. Using polymorphic SSR molecular markers covering the 12 rice chromosomes, the candidate gene was initially mapped between markers LS4 and LS6 on the short arm of chromosome 1 (**Figure 5A**). To identify the target gene, we designed four pairs of molecular markers between LS4 and LS6. The candidate gene was ultimately mapped within a 123 kb interval between Indel8-3 and Indel8-4 (**Figure 5A**). This interval contains 17 annotated genes. Only the coding sequence of *LOC_Os01g18320* contained a mutation: from ‘T’ to ‘G’ at nucleotide 437, changing amino acid 146 from Leu to Arg (**Figure 5A**). *LOC_Os01g18320*, encoding a PPOX protein.

*LOC_Os01g18320*, encoding a PPOX. Since PPOX converts protoporphyrinogen IX to protoporphyrin IX, the content of protoporphyrin IX production will be reduced when PPOX synthesis is defective. As determined, the content of protoporphyrin IX was 0.18 μg/g in the wild type and 0.08 μg/g in the mutant (**Figure 5B**). Thus, the protoporphyrin IX content varied significantly. This suggests a defect in protoporphyrin IX synthesis in the *lmm8* mutant significant.

To further test whether *LOC_Os01g18320* corresponds to the *LMM8* gene, we amplified a 6342 bp fragment of this gene from wild type genomic DNA and generated a complementation vector, which we introduced into the *lmm8* mutant by *Agrobacterium tumefaciens*-mediated transformation. We obtained 23 transgenic complementation plants. The lesion phenotype and all other mutant traits of *lmm8* were fully restored to wild type phenotypes in the complementation plants (**Figure 5D**; **Supplementary Data 3**). By sequencing the mutant sites of WT, *lmm8* mutant and T_0_ transgenic plants, only the transgenic plants showed polymorphism at the mutant sites (both T and G), which confirmed that the transformation was successful (**Figure 5C**). Therefore, the *LMM8* gene is *LOC_Os01g18320*, encoding a PPOX.

Amino acid sequence alignment of LMM8-homologs showed that PPOX proteins are highly conserved in different species (**Figure S2**). We built a phylogenetic tree based on the homologous protein sequences, confirming that PPOX is widely present in different organisms. LMM8 in rice is most closely related to PPOX in maize (*Zea mays*), followed by *Sorghum bicolor*. Two PPOX proteins are present in *Arabidopsis thaliana*, and PPOX in rice is related to PPO1 (*NP192078.1*) in *Arabidopsis thaliana*, but not to PPO2 (*NP568717.2*) (**Figure S3**).

### 3.5 Expression analysis of *LMM8*

**Figure 6:**
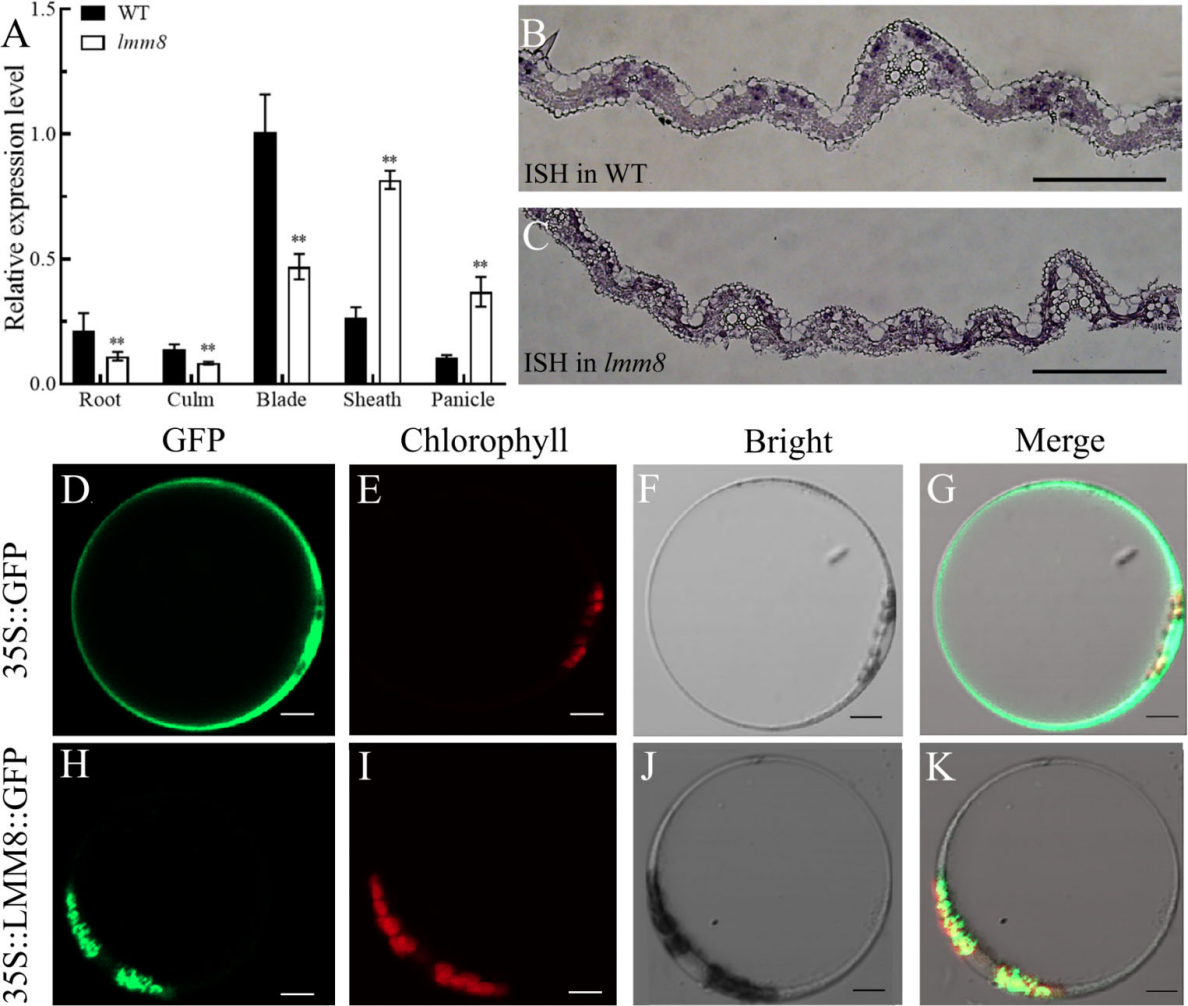
LMM8 expression pattern analysis **(A)** RT-qPCR assay of *LMM8* in wild type and *Imm8* mutant tissues; values represent means + SD(n = 3) (*: *P* < 0.05, **: *P* < 0.01) **(B)** *In situ* hybridization of *LMM8* mRNA in wild type leaves; **(C)** *In situ* hybridization of *LMM8* mRNA in *Imm8* leaves; scale bars =2mm: **(D-G)** Subcellular localization of the pAN580 in rice protoplasts; **(H-K)** Subcellular localization of the pAN580-LMM8 in rice protoplasts: scale bars=5μm.

To analyze the expression pattern of *LMM8*, we performed RT-qPCR. *LMM8* was expressed in root, culm, blade, sheath, and panicle in both wild type and *lmm8* plants. This gene was expressed at significantly lower levels in roots, culms, and blades of *lmm8* compared to the wild type but at higher levels in sheath and panicle (**Figure 6A**). To analyze *LMM8* expression in leaves, we performed *in situ* hybridization of *LMM8* mRNA in wild type and *lmm8* leaves. The DIG signal was mainly concentrated in mesophyll cells, and no ectopic expression was detected (**Figures 6B, C**). To explore the subcellular localization of LMM8, we transformed rice protoplasts with a recombinant vector expressing LMM8-GFP fusion protein. The GFP signal observed in protoplasts transformed with empty vector was distributed throughout the protoplast except for the vacuole, as determined by confocal microscopy (**Figures 6D–G**). By contrast, the GFP signal from LMM8-GFP fusion protein overlapped with red autofluorescence produced by chloroplasts, indicating that LMM8 functions in chloroplasts (**Figures 6H–K**).

### 3.6 The response of *lmm8* lesions to light

**Figure 7:**
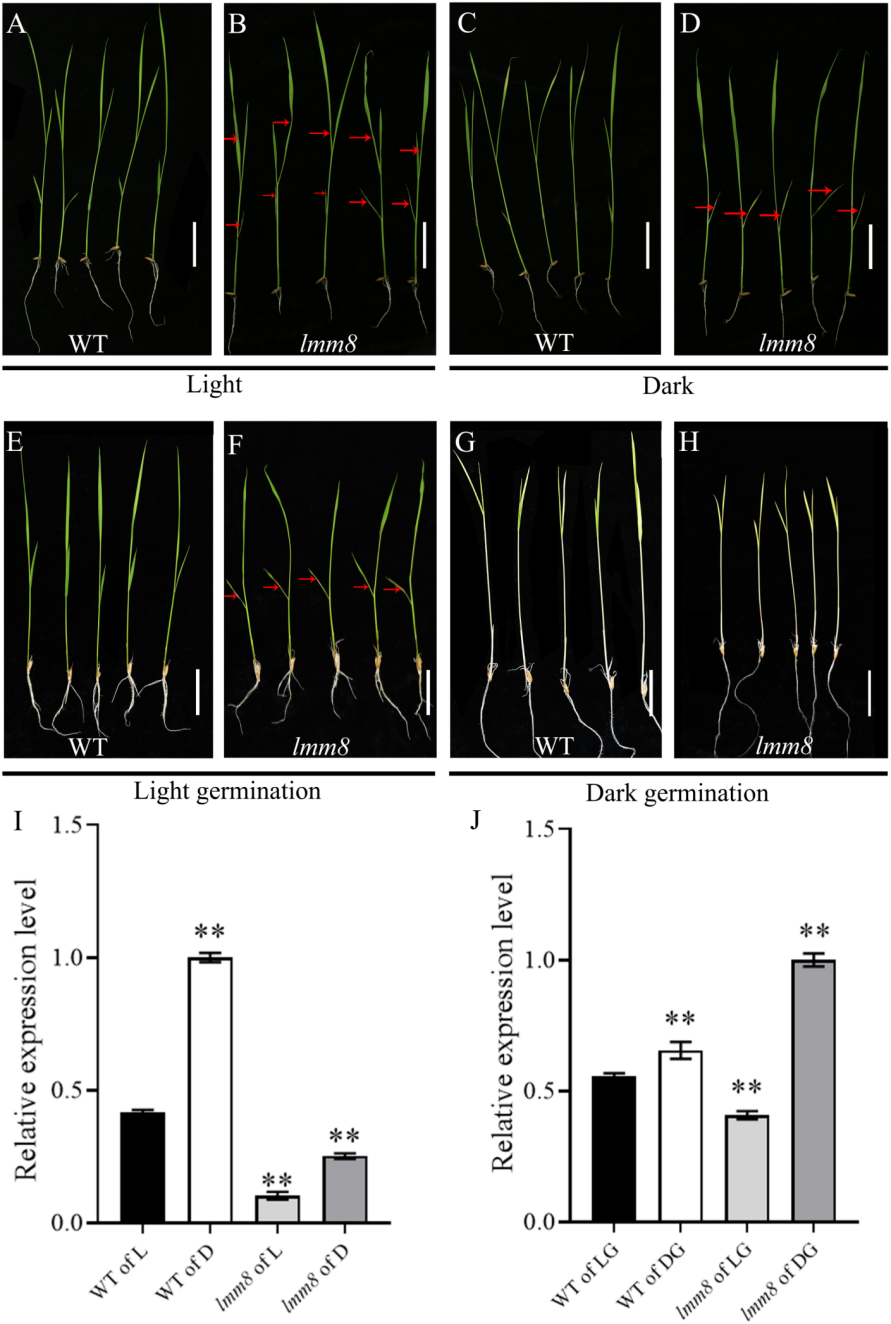
The light and darkness treatment of wild type and *Imm8* mutant **(A, B)** Wild type and *Imm8* mutants grown under light: **(C, D)** Wild type and *Imm8* mutants treated with whole plant shading from the light stage to the second leaf stage; **(E, F)** Wild type and *Imm8* mutants germinated to second leaf stage under light; **(G, H)** Wild type and *Imm8* mutants germinated to second leaf stage under dark; scale bars =2cm, (red arrows indicate the location of the diseased spots): **(I)** Relative expression levels of *LMM8* under light (L) and dark (D); **(J)** Relative expression levels of *LMM8* under light germunation (LG) and darkgermunation (DG), values represent means ± SD (n=3): (*: *P* < 0.05, **: *P* < 0.01).

Spot formation in many lesion-mimic mutants is induced by light exposure. Therefore, we performed a series of shading treatments. When we treated wild type and *lmm8* plants at the second-leaf stage with shade, no lesions were detected in shaded or unshaded wild type leaves (**Figures 7A, C**). However, in the *lmm8* mutant, the brown lesions of unshaded leaves spread from the bottom leaf to the second leaf (**Figure 7B**). By contrast, lesions did not spread in shade-treated *lmm8* plants (**Figure 7D**). When wild type and *lmm8* seeds were germinated and the plants grown to the second-leaf stage in the light or dark, wild type seeds germinated well under both conditions and the plants failed to produce lesions on their leaves (**Figures 7E, G**). *lmm8* seeds also germinated well in the light but showed some lesions on the lowest leaves (**Figure 7F**). In the dark, the *lmm8* mutant also germinated well but showed no lesions on their lowest leaves (**Figure 7H**). We later analyzed the expression level of *LMM8* from each treatment by RT-qPCR. The results showed that the expression level of *LMM8* increased very significantly after both the dark treatment and the dark germination treatment (**Figures 7I, J**).

### 3.7 Analysis of disease resistance in *lmm8*

In total, 108 effective rice blast strains have been identified in seven Chinese varieties, which were divided into six Chinese physiological groups and 26 physiological species, including the dominant ZB population (69.44% occurrence frequency) and the important ZA population (12.96% occurrence frequency; **Supplementary Data 4**). The resistance spectra of lines with twenty-four single rice blast genes in six Chinese physiological populations are shown in **Supplementary Data 5**.

We inoculated wild type and *lmm8* plants at the seedling stage with six Chinese physiological groups (ZA, ZB, ZC, ZD, ZE, ZG) of rice blast strains and analyzed the resistance of the plants. Compared to the wild type, the resistance frequency of *lmm8* to the dominant population ZB was 57.33% higher, and its resistance frequency to the important population ZA was 42.86% higher. For the ZC and ZD populations, the *lmm8* mutant also showed significantly higher resistance frequency than the wild type. The wild type and *lmm8* mutant showed similar resistance frequencies to the ZE and ZG populations. The total disease resistance frequency of *lmm8* was 77.78%, i.e., nearly 53.71% higher than that of the wild type, and the putative resistance spectrum to rice blast strains was significantly broader in the *lmm8* mutant than the wild type (**Table 1**).

**Table 1:** Resistance Spectral determination of the wild type and *lmm8*.

Materials	Resistance frequency to each physiological group (%)						Resistance frequency to total population (%)
	ZA	ZB	ZC	ZD	ZE	ZG	
WT	7.41	22.67	25.00	0.00	100.00	100.00	24.07
*lmm8*	50.00	80.00	87.00	50.00	100.00	100.00	77.78

**Figure 8:**
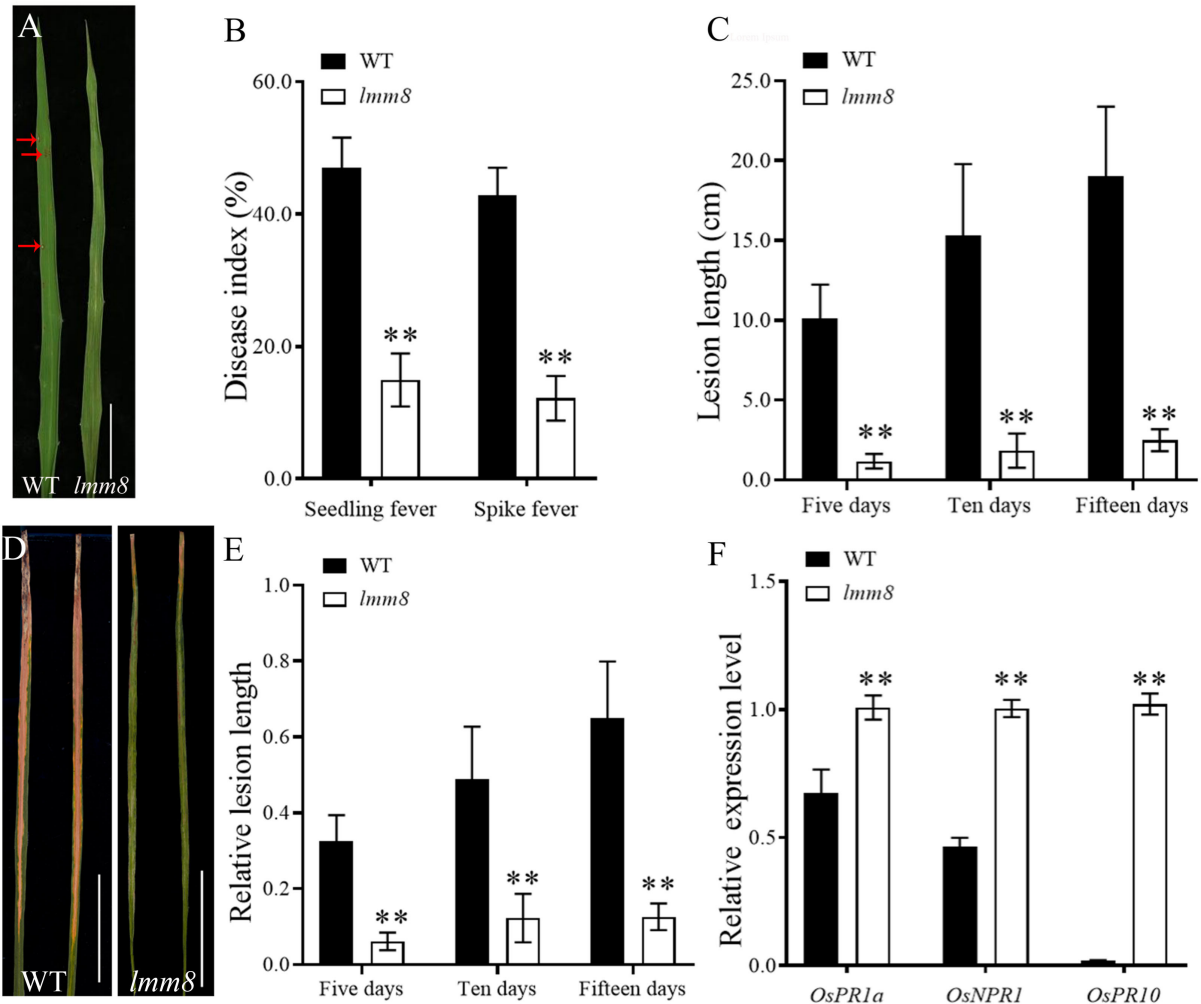
Resistance identification of wild type and *Imm8* mutant **(A)** Leaf phenotype of wild type and *Imm8* mutant after inoculation with rice blast, scale bar= 5cm **(B)** Disease index of seedling blast and spike blast of wild type and Imm8 mutant after inoculation with rice blast, values represent means ± SD (n = 15) **(C)** Leaves of wild type and *Imm8* mutant inoculated with bacterial blight for 15 days (n = 15) ; scale bars =5cm; **(D)** Length of diseased spots in wild type and *Imm8* mutant leaves after 5, 10 and 15 days inoculation with bacterial blight. **(E)** Relative spot length of wild type and *Imm8* mutant leaves 5, 10 and 15 days inoculation with bacterial blight (n = 15) : **(F)** Expression of genes involved in immune defense in the normal wild type and *Imm8* mutant forms; (n = 3) : (*: *P* <0.05, **: *P* < 0.01).

Analysis of the resistance to seedling fever and spike fever of wild type and *lmm8* showed that the disease index of *lmm8* mutant was significantly lower than that of the wild type (**Figures 8A, B**). We inoculated wild type and *lmm8* leaves with bacterial blight at the beginning of tillering. The spot length and relative spot length were significantly lower in the *lmm8* mutant than the wild type 5, 10, and 15 days after inoculation (**Figures 8C–E**). Expression analysis of immune defense-related genes in the wild type and *lmm8* showed that the disease-related gene *OsPR1a*, *OsPR10* and *OsNPR1* were significantly activated in *lmm8* mutant (**Figure 8F**). Therefore, the *lmm8* mutant exhibited increased disease resistance and resistance to a broader spectrum of rice blast strains than the wild type.

## 4 Discussion

Lesion-mimic mutants exhibit necrotic lesions of different colors and sizes on the leaf, leaf sheath, or stalk, that are produced spontaneously in the presence or absence of external factors (Han et al., 2014). Lesion-mimic mutants can be divided into three types based on the time of lesion appearance: WLLM (whole-life lesion mimics), VILM (vegetative-initiation lesion mimics), and RILM (reproductive initiation lesion mimics) (Jiao et al., 2018). These mutants can also be divided into two types based on the lesion formation pattern: initial type and extended type (DangI et al., 1996). The *lmm8* mutant isolated in the current study showed lesion-mimic spots on leaves from the second-leaf stage to maturity. The lesion-mimic spots in *lmm8* gradually spread from the second leaf to all leaves. Therefore, the *lmm8* mutant is an extended-type and whole-life lesion mimic.

Many rice lesion-mimic mutants show adverse effects on agronomic traits (Lin et al., 2022). In this study, the *lmm8* mutant showed significantly reduced plant height at the second-leaf stage, along with a significantly reduced internode length and width. Cell length and cell width at the seedling stage were also significantly lower than the wild type. Finally, the plant height, panicle length, grain number per panicle, seed setting rate, effective panicle number, 1000-grain weight and grain length were significantly reduced in the *lmm8* mutant. The dwarfism of the *Oryza sativa dwarfing broad-leaf mutant 1* (*osdwl1*) mutant is due to a shorter internode length and reduced cell length (Huang et al., 2021), and the dwarfism of *dwarf and curled flag leaf 1* (*dcfl1*) is due to reduced cell length (Zhang et al., 2017). The *lmm8* mutant exhibited a reduced cell size. These findings indicate that LMM8 positively regulates plant type in rice and plays an important role in determining plant type. This could be another function of the *LMM8*.

Substantial PCD appeared in the leaves of *lmm8*. Several major PCD pathogenesis pathways have been identified in plants, such as pathways involving (i) chloroplasts and light energy; (ii) sphingolipids and fatty acids; (iii) signal perception at the plasma membrane, which requires efficient membrane trafficking; (iv) secondary messengers such as ion fluxes and ROS; and (v) the control of gene expression as the last integrator of signaling pathways (Bruggeman et al., 2015). We detected reduced photosynthetic pigment contents and chloroplast degradation in the *lmm8* mutant, indicating that the lesion-mimic spots in *lmm8* are closely related to chloroplast degradation. Moreover, the H_2_O_2_ content in *lmm8* leaves was 1.8-times higher than that of the wild type, and CAT, POD, and SOD activities were significantly reduced, indicating that large amounts of ROS accumulate in *lmm8*. High ROS levels in plant cells can damage macromolecular substances and other components in the cell, which can lead to cell death and hinder normal metabolism and plant growth (Xue et al., 2013). Therefore, the strong accumulation of ROS in *lmm8* is also responsible for its PCD and lesion-mimic spots. Finally, spot formation is induced by light in some lesion-mimic mutants and induced by dark in others (Wang et al., 2017; Cui et al., 2021; Du et al., 2021; Ren et al., 2022). The appearance and production of lesion-mimic spots in *lmm8* was induced by light, and the expression level of *LMM8* decreased. Therefore, *lmm8* is a novel light-regulated lesion mimetic mutant and *LMM8* expression is regulated by light.

*LMM8* is the *LOC_Os01g18320* gene, encoding a PPOX involved in tetrapyrrole biosynthesis. *LOC_Os01g18320* is related to the previously reported gene *Spotted and Rolled Leaf 1* (*SPRL1*). *LOC_Os01g18320* in the *sprl1* mutant contains a base substitution from ‘C’ to ‘T’ at nucleotide 516, causing an amino acid change from ‘Arg’ to ‘Trp’ (Liu et al., 2022). The *sprl1* and *lmm8* mutants have some differences in phenotype. For example, the *sprl1* mutant exhibits lesion-mimic spots throughout plant growth, and its leaves curl inward during the third-leaf stage (Liu et al., 2022), whereas *lmm8* has a more severe lesion-mimic phenotype, with off-white lesions at the third-leaf stage. Unlike the *sprl1* mutant, *lmm8* showed no leaf curling. These phenotypic differences might be related to the different mutation sites in *sprl1* and *lmm8*. Indeed, different mutation sites in a single gene can lead to different phenotypes (Wang et al., 2015; Zheng et al., 2021).

Most rice lesion-mimic mutants reported to date show increased disease resistance (Qian et al., 2021). The *lmm8* mutant showed enhanced resistance to bacterial blight and rice blast and resistance to a broad spectrum of rice blast strains. The accumulation of ROS, callose, and other resistance-related substances in plants invaded by pathogens is a natural defense response that prevents the transfer of nutrients from the host to pathogen, delays pathogen growth, and activates the host defense system (Zhang et al., 2021). For example, the *spl28* (*spotted leaf 28*) mutant accumulates high levels of ROS, callose, phytoantitoxin, and other resistance-related substances around the disease spots, significantly enhancing resistance to bacterial blight and rice blast (Qiao et al., 2010). When the *Oryza Sativa Natural Resistance-associated Macrophage Protein 1* (*OsNRAMP1*) gene was mutated, the plants showed significantly increased ROS accumulation and increased resistance to bacterial blight, bacterial stripe, rice blast, and false smut (Chu et al., 2022). *OsPR1a* (*pathogenesis-related protein 1a*), is an important part of the plant defense system, which is often activated in the plant disease resistance or stress resistance (Li et al., 2012). *OsNPR1*(*a non-expressor of pathogenesis-related genes1*), can use it to activate a specific and appropriate defense response against invaders by modulating signaling pathways (Kothari et al., 2016). And *OsPR10* (*Orysa sativa pathogenesis-related protein 10a*), mediating the defense response through a direct interaction with the transcription factor OsWRKY19 in the nucleus (Du et al., 2021). ROS highly accumulated in the *lmm8* mutant, and the disease-related genes *OsPR1a*, *OsNPR1* and *OsPR10* were significantly activated in the mutant. This suggests that *LMM8* plays a role in disease resistance. Therefore, LMM8 balances plant development and disease resistance in rice. Because *lmm8* mutants show stronger broad-spectrum resistance to rice blast and bacterial blight, it could be used as strain identification varieties in rice disease resistance breeding. And in the future, *lmm8* can also be used to cross with other rice varieties to aggregate the excellent resistance of *lmm8*.

## 5 Conclusions

In conclusion, *LMM8* encodes a key PPOX enzyme involved in tetrapyrrole biosynthesis in rice. The loss of function of LMM8 in the *lmm8* mutant led to lesion mimic and dwarf phenotypes. And the mutant showed that ROS hyperaccumulation, programmed cell death and enhanced resistance. Our findings suggest that LMM8 plays an important role in regulating plant type and defense responses in rice. Together, our results provide new insight on the function of the LMM8 protein.

## Data availability statement

The datasets presented in this study can be found in online repositories. The names of the repository/repositories and accession number(s) can be found in the article/**Supplementary Material**.

## Author contributions

CZ, GH, MZ, YG and HS designed the experiments. MZ, YG, HS, JD, XP, LZ, XW, HY, YQ and XL performed the experiments. MZ, YG, HS, YQ and XL analyzed the data. MZ, YG and CZ wrote the manuscript. MZ, LZ, YG, and CZ revised the manuscript. All authors contributed to the article and approved the submitted version.
