# Supplementary material for: Lesion mimic mutant 8 balances disease resistance and growth in rice
Source: Front Plant Sci. 2023 Jun 5;14:1189926. doi: 10.3389/fpls.2023.1189926 (PMC10278592; doi:10.3389/fpls.2023.1189926)
Supplement: Supplementary file 1 [file DataSheet_1.pdf]

Supplementary Material

Lesion Mimic Mutant 8 balances disease resistance and growth in rice

Changwei Zhang*, Mengcan Zhao, Yunxia Guo, Hang Sun

* **Correspondence**: Corresponding Author: zcw2013@swu.edu.cn

Supplementary Data 1. All primer sequences used in this study

Purpose	Primer name	sequence
Mapping	RM493F	GTACGTAAACGCGGAAGGTGACG
	RM493R	CGACGTACGAGATGCCGATCC
	LS2-F	CGCAAGAACTAAGCCAACCAGA
	LS2-R	AAGGCGACAGGAGCTGATCTC
	LS4-F	CAGGGTTCACAGGAGGATTCAAC
	LS4-R	AGATCCAGGTCACCGACGATG
	LS6-F	AGCACATGGTTGTGAGACTGTTTG
	LS6-R	CTAGGTTAGCCGTTTGCGGA
	LS7-F	CGTTGTTCCTGTCCATGAGGTT
	LS7-R	CTCCTTCACAGCGAACGGTTAGA
	LS10-F	TGTATTGGTATATGTGTAGGATAG
	LS10-R	GATGATGATCGAAAGCTGTAGTAGAG
	Indel8-2-F	TCCTGTAACTTCTTTCGGATTTCC
	Indel8-2-R	GCCTCCTCAATTTGCTATCAGG
	Indel8-3-F	GGGAATGGCTATTAGTGTGTAGACC
	Indel8-3-R	CACACACCATGTGTGTCCTCACC
	Indel8-4-F	TGAAGTGGTCATTGGTTCGATTC
	Indel8-4-R	CACACACCATGTGTGTCCTCACC
	Indel8-5-F	GCATCGGCCATTCAGTTCAT
	Indel8-5-R	AGGTGCTTGAGCCTTTGTGC
Complementation test	F1	CTAGTCATTCATACAACCCGCAGC
	R1	TGCATCAAATCCCTCCACTGC
	COM-LMM8-F	GTTTGGTGTTACTTCTGCAGGATCC CTAGTCATTCATACAACCCGCAGC
	COM-LMM8-R	ACTTACCGTCGACCTCGAGGGTACC TGCATCAAATCCCTCCACTGC
RT-qPCR	LMM8-F	CTTTCCAAATCGTGCTCCAGC
	LMM8-R	CCAATGAGGAACTGTGGTATGGC
	OsWRKY45-F	AATTCGGTGGTCGTCAAG
	OsWRKY45-R	TTTGGGTGCTTGGAGTTT
	OsPR1a-F	GGTACGACCACGGCAGCAAC
	OsPR1a-R	GATTGGCCGACGAAGTTGC

	OsNPR1-F	AGGTTGCTGTATCTTGAA
	OsNPR1-R	AATATCCATTGCTACTCTTG
	OsPR10-F	CGGAGAAGGAGAAGGACATC
	OsPR10-R	GGTGAGCGACGAGGTAGT
	Action-F	GACCCAGATCATGTTTGAGACCT
	Action-R	CAGTGTGGCTGACACCATCAC
In situ hybridization	LMM8-374-F	CACCGCCACCTCCGCCAC
	LMM8-374-R	AGATTTAGGTGACACTATAGAA GATCGTCCTTGAGCCCGCTGT
Subcellular localization	LMM8-GFP-F	TCTTAAGTCCGGAGCTAGCTCTAGA ATGGCCGCCGCCG
	LMM8-GFP-R	CCTCGCCCTTGCTCACCATGGATCC CTTGTAGGCGTACTTGGTCAA

Supplementary Data 2. Species and numbers for analysis were obtained from NCBI

Species name	number
Oryza sativa Japonica Group	>NP_001388666.1
	>BAD81569.1
Oryza glaberrima	>XP_052140608.1
Eleusine indica	>QFB70735.1 (*LOC_Os01g18320*)
Zea mays	>NP_001105564.2
Sorghum bicolor	>XP_002455484.1
Gossypium raimondii	>XP_012490260.1
Populus trichocarpa	>XP_002302810.1
Arabidopsis thaliana	>NP_192078.1 (PPO1)
	>NP_568717.2 (PPO2)
Homo sapiens	>NP_000300.1
Mus musculus	>NP_032937.1
Hordeum vulgare	>KAE8789512.1
Triticum aestivum	>KAF7056819.1

Supplementary Data 3. Statistics of agronomic traits of the wild type, *lmm8* and COM-*lmm8*

Materi al	Plant eight(cm)	Effective panicle	Panicle length(cm)	Number of seeds per panicle	Seed setting rate (%)	1000-grain weight (g)
WT	84.54±1.98	5.90±1.10	21.60±2.55	87.50±5.19	89.65±3.30	22.89±0.54
lmm8	58.81±2.24**	4.70±0.95*	16.60±1.69**	43.75±3.14**	83.05±2.73**	21.00±0.27*
COM-*lmm8*	79.71±2.45	5.40±0.80	21.33±1.36	76.35±4.77*	88.45±2.44	22.32±0.27

values represent means ± SD (n = 10);(* : $P < 0.05$, ** : $P < 0.01$) .

Supplementary Data 4. Number of effective strains tested, number of physiological races and occurrence frequency of physiological races

No. of effective isolates	No. of races	Occurrence frequency of physiological group (%)					
		ZA	ZB	ZC	ZD	ZE	ZG
108	26	12.96	69.44	7.41	1.85	0.93	7.41

Supplementary Data 5. Determination of the resistance spectrum of rice blast single gene lines

Strain or variety name	Resistance gene	Disease resistance frequency（%）						Disease resistance frequency (%）
		ZA	ZB	ZC	ZD	ZE	ZG	
IRBL11-Zh	*Pi11*	0	9.52	42.86	0	0	0	12.96
IRBLa-A	*Pia*	0	16.67	28.57	100	0	50.00	20.37
IRBL20-IR24	*Pi20*	0	4.76	28.57	0	0	0	7.41
IRBL12-M	*Pi12*	0	11.90	14.29	0	0	0	11.11
IRBLt-K59	*Pit*	0	4.76	0	0	0	0	3.70
IRBL19-A	*Pi19*	0	28.57	57.14	0	0	50.00	31.48
IRBLz5-CA	*Piz5*	0	52.38	57.14	100	100	50	53.70
IRBL3-CP4	*Pi3*	0	7.14	0	0	0	0	5.56
IRBLta2-Pi	*Pita2*	100	85.71	100	100	100	100	90.74
IRBLks-F5	*Piks*	0	9.52	14.29	0	0	50.00	11.11
IRBLz-Fu	*Piz*	0	16.67	14.29	0	0	50.00	16.67
IRBL1-CL	*Pi1*	0	59.52	85.71	0	0	100	61.11
IRBLk-Ka	*Pik*	0	59.52	57.14	0	0	50.00	53.70
IRBLkh-K3	*Pikh*	100	66.67	85.71	0	0	100	68.52
IRBLta-K1	*Pita*	0	64.29	14.29	100	0	50.00	64.81
IRBL7-M	*Pi7*	0	61.90	57.14	0	0	100	61.11
IRBLzt-T	*Pizt*	0	16.67	14.29	0	0	50.00	16.67
IRBLkp-K60	*Pikp*	0	69.05	71.43	0	0	50.00	64.81
IRBLb-B	*Pib*	0	7.14	0	0	0	0	5.56
IRBLkm-Ts	*Pikm*	0	69.05	85.71	0	0	50.00	66.67
IRBLi-F5	*Pii*	100	52.38	57.14	100	100	0	53.70
IRBL9-W	*Pi9*	0	50.00	42.86	100	100	100	51.85
IRBL5-M	*Pi5*	0	28.57	71.43	0	100	100	37.04
IRBLsh-B	*Pish*	0	7.14	0	0	0	0	7.41
Tetep（CK）		0	100	100	100	100	100	100

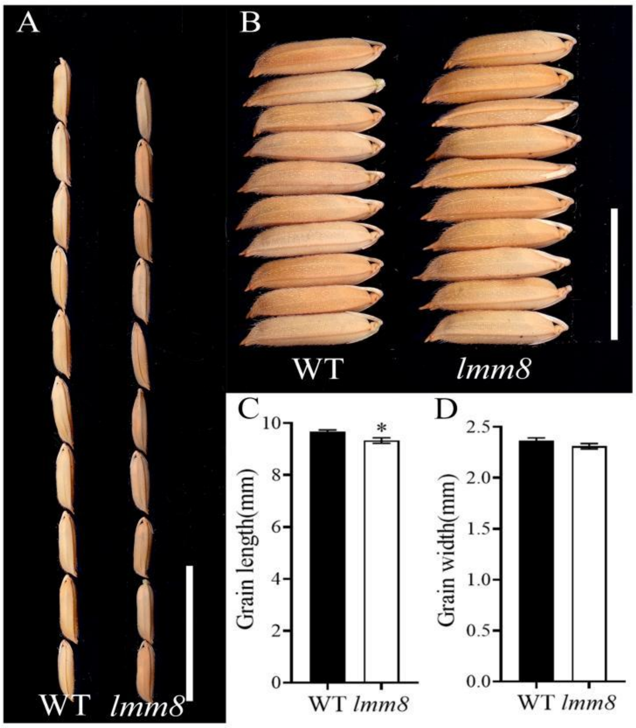

Fig.S1.Comparison of grain length and width between wild type and *lmm8* mutant

A: Comparison of wild type and *lmm8* mutant seed length,scale bar=2cm; B: Comparison of wild type and *lmm8* mutant seed width, scale bar=1cm; C: Grain length of wild type and *lmm8* mutant; D: Grain width of wild type and *lmm8* mutant; values represent means ± SD (n = 3); (* : P < 0.05, * * : P < 0.01) .

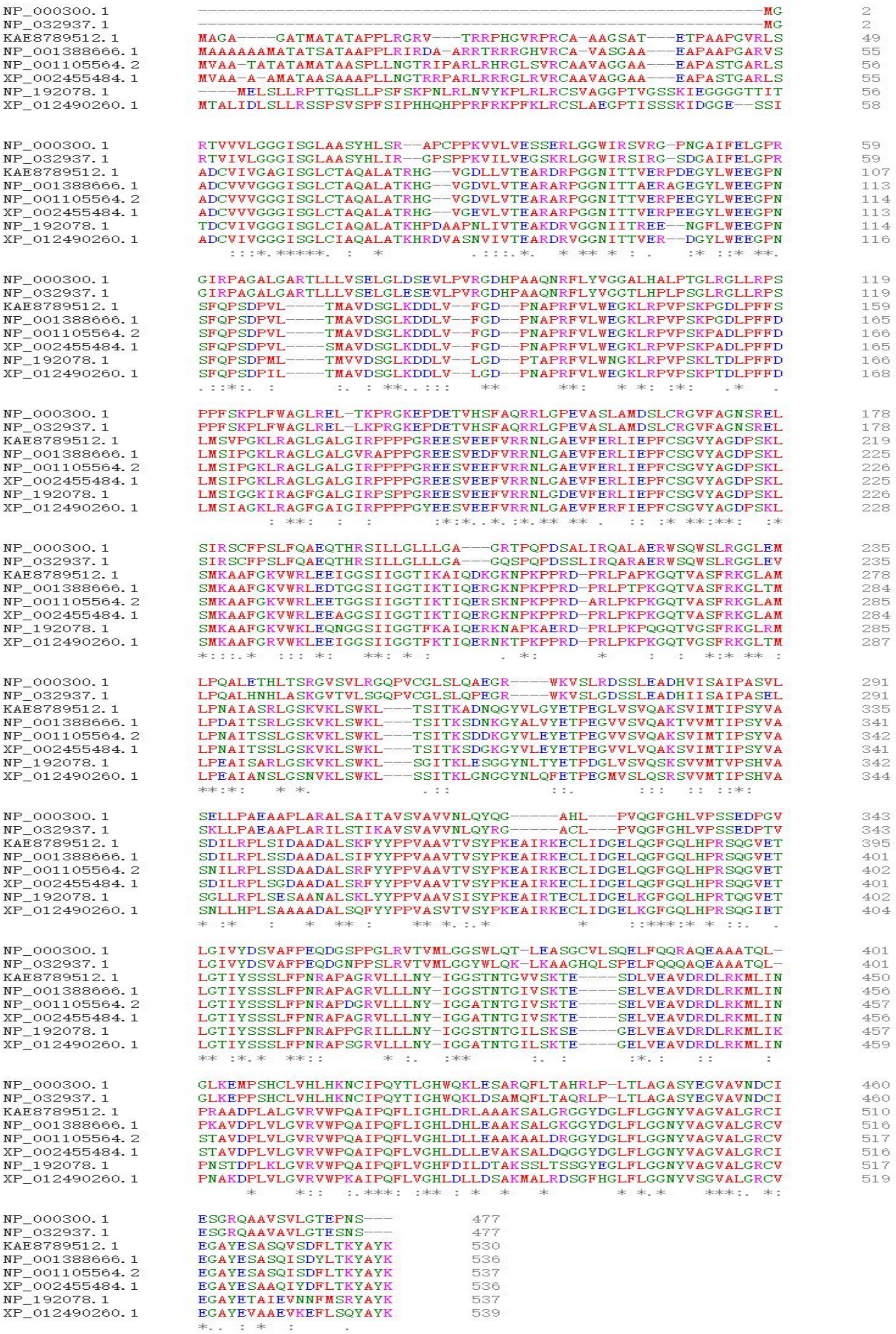

Fig.S2. Attached is Figure 2 Sequence alignment of PPOX in different organisms.

NP_000300.1 （*Homo*); NP_032937.1(*Mus musculus*);KAE8789512.1(*Hordeum vulgare*); NP_ 001388666.1(*Oryza sativa Japonica Group*); NP_001105564.2(*Zea mays*); XP_002455484.1 (*Sorghum bicolor*); NP_192078.1(*Arabidopsis thaliana*); XP_012490260.1(*Gossypium raimondii*).

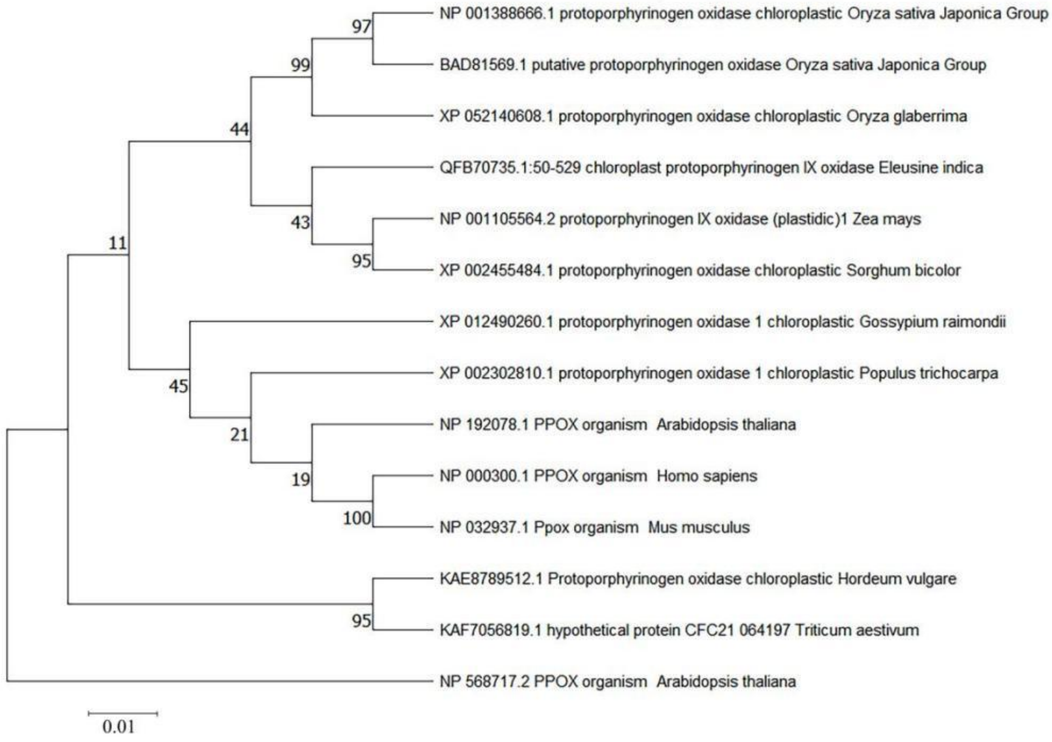

Fig.S3. Attached is Figure 1 Phylogenetic analysis of PPOX in different organisms.
