# Supplementary material for: Lesion mimic mutant 8 balances disease resistance and growth in rice
Source: Front Plant Sci. 2023 Jun 5;14:1189926. doi: 10.3389/fpls.2023.1189926 (PMC10278592; doi:10.3389/fpls.2023.1189926)
Supplement: Supplementary file 2 [file DataSheet_2.pdf]

Species and numbers for analysis were obtained from NCBI

Species name	number
Oryza sativa Japonica Group	>NP_001388666.1
	>BAD81569.1
Oryza glaberrima	>XP_052140608.1
Eleusine indica	>QFB70735.1 (*LOC_Os01g18320*)
Zea mays	>NP_001105564.2
Sorghum bicolor	>XP_002455484.1
Gossypium raimondii	>XP_012490260.1
Populus trichocarpa	>XP_002302810.1
Arabidopsis thaliana	>NP_192078.1 (PPO1)
	>NP_568717.2 (PPO2)
Homo sapiens	>NP_000300.1
Mus musculus	>NP_032937.1
Hordeum vulgare	>KAE8789512.1
Triticum aestivum	>KAF7056819.1
